# Secretome of Intestinal *Bacilli:* A Natural Guard against Pathologies

**DOI:** 10.3389/fmicb.2017.01666

**Published:** 2017-09-01

**Authors:** Olga N. Ilinskaya, Vera V. Ulyanova, Dina R. Yarullina, Ilgiz G. Gataullin

**Affiliations:** ^1^Department of Microbiology, Kazan Federal University Kazan, Russia; ^2^Department of Surgery and Oncology, Regional Clinical Cancer Center Kazan, Russia

**Keywords:** *Lactobacillus*, *Bacillus*, gastrointestinal tract, metabolites, secretome

## Abstract

Current studies of human gut microbiome usually do not consider the special functional role of transient microbiota, although some of its members remain in the host for a long time and produce broad spectrum of biologically active substances. Getting into the gastrointestinal tract (GIT) with food, water and probiotic preparations, two representatives of *Bacilli* class, genera *Bacillus* and *Lactobacillus*, colonize epithelium blurring the boundaries between resident and transient microbiota. Despite their minor proportion in the microbiome composition, these bacteria can significantly affect both the intestinal microbiota and the entire body thanks to a wide range of secreted compounds. Recently, insufficiency and limitations of pure genome-based analysis of gut microbiota became known. Thus, the need for intense functional studies is evident. This review aims to characterize the *Bacillus* and *Lactobacillus* in GIT, as well as the functional roles of the components released by these members of microbial intestinal community. Complex of their secreted compounds is referred by us as the “bacillary secretome.” The composition of the bacillary secretome, its biological effects in GIT and role in counteraction to infectious diseases and oncological pathologies in human organism is the subject of the review.

## Introduction

The human gut microbiota consists of about 1500 microbial species which constitute 10^12^ bacteria per gram of stool (Browne et al., 2016). The gastrointestinal microbiota of healthy human adults consists primarily of bacteria belonging to phyla *Firmicutes* and *Bacteroidetes*, and to a lesser extent to phyla *Actinobacteria* and *Proteobacteria* (Dethlefsen et al., 2008; Yang and Jobin, 2014). Density and composition of microbiota varies along both the length of the gut and the cross-section (Nava and Stappenbeck, 2011; Tropini et al., 2017). Changes in nutrients, availability of oxygen, and presence of immune effectors in local microenvironment determine species variation and abundance (Donaldson et al., 2016). The most dominant taxa have the highest stability in the gut (Martí et al., 2017). Rapidly dividing facultative anaerobes from *Lactobacillaceae* and *Enterobacteriaceae* dominate in small intestine, while saccharolytic representatives of *Bacteroidales* and *Clostridiales* orders are abundant in the large intestine (Donaldson et al., 2016; Tropini et al., 2017). Mucin-utilizing species of *Akkermansia* and *Bacteroides* are followed by aerotolerant *Proteobacteria* and *Actinobacteria* in direction to the epithelium (Tropini et al., 2017). *Proteobacteria* and *Firmicutes* are found in crypts and represent the stock for reseeding the colon because they are protected from the luminal flow (Tropini et al., 2017). At least 50–60% of the bacterial genera from the intestinal microbiota of a healthy individual produce spores which facilitate both microbiota persistence and transmission (Browne et al., 2016). The majority of gut bacteria are transient populations which pass through the lumen of the lower GIT (Tropini et al., 2017).

Composition of gut microbiota varies among individuals with geographic provenance, gender, age, diet, malnutrition, and intake of probiotics or antimicrobial agents (Panda et al., 2014; Haro et al., 2016; Odamaki et al., 2016; Maffei et al., 2017; Martí et al., 2017; Singh et al., 2017). Alterations in the composition of the gut microbiota and reductions in microbial diversity lead to different disorders such as inflammatory conditions of the intestine (inflammatory bowel disease, irritable bowel syndrome, colorectal cancer) (Gagnière et al., 2016; Ghoshal et al., 2017; Rapozo et al., 2017), type 2 diabetes, obesity, anorexia nervosa, forms of severe acute malnutrition, cardiovascular diseases (atherosclerosis, hypertension, heart failure) (Tang et al., 2017), neurobehavioral diseases (autism spectrum disorder, major depression) (Clemente et al., 2012). The microbiota is increasingly recognized for its ability to maintain homeostasis in health and disease influencing host appetite, function of the nervous system and several complex host behaviors (Sharon et al., 2016; Winek et al., 2016; van de Wouw et al., 2017; Vuong et al., 2017). A healthy gut microbiota can be defined by the presence of the various microbial species that enhance metabolism, resistance to infection and inflammation, prevention against cancer and autoimmunity.

Lactobacilli are historically considered as integral part of human intestinal microbiota. Today, a large body of evidence indicates that only a small number of *Lactobacillus* species are true autochthonous inhabitants of the mammalian intestinal tract and that most lactobacilli present are allochthonous members derived from food or oral cavity (Reuter, 2001; Walter, 2008). *Lactobacillus* spp. content of fecal samples is characterized by temporal fluctuations and lack of stability (Walter et al., 2001; Vanhoutte et al., 2004). Attempts to divide lactobacilli into resident and transient ones are hardly legitimate since the style of nutrition significantly affects their contents in the intestine. Lactobacilli together with enterococci dominate in the duodenum and in the jejunum (Reuter, 2001; Hammes and Hertel, 2015), although their absolute number increases along the intestine from duodenum to colon (Derrien and van Hylckama Vlieg, 2015). However, they constitute only a minor fraction within the human adult fecal microbiota, i.e., around 0.01 to 0.6% of total bacterial counts (Harmsen et al., 2002; Matsuda et al., 2009).

Metagenomics widely used for study of gut microbiota is unable to detect bacteria at concentrations less than 10^5^ bacteria per gram (Lagier et al., 2015). A culturing approach that uses high-throughput culture conditions in combination with matrix-assisted laser desorption/ionization-time of flight (MALDI-TOF) mass spectrometry and 16S rRNA sequencing for taxonomic identification and referred to as culturomics has allowed significant increase in a number of bacteria discovered in human GIT including species known in humans but not in the gut, species previously considered unculturable as well as new species. Considerable part of them is represented by *Firmicutes* (Lagier et al., 2016) including *Bacillus* species (Lagier et al., 2015; Mourembou et al., 2016; Senghor et al., 2017).

For a long time, the representatives of *Bacillus* genus, unlike the species of *Lactobacillus*, were not considered as a part of the normal intestinal microbiome. Being isolated from feces, *Bacillus* spp. as soil microorganisms were considered transient. Recent studies show that they are present in the GIT in the amounts significantly higher than what can be explained by their ingestion with food only. *Bacillus* spp. (*B. pumilus, B. licheniformis, B. clausii, B. subtilis, B. megaterium, B. mediterraneensis*, *B. thuringiensis*) have been isolated from the healthy human GIT, where they are well-adapted and are more colonizing than transient (Fakhry et al., 2008; Alou et al., 2016; Lopetuso et al., 2016). In environment, the vegetative forms of *Bacillus* are present usually near decomposing plants and in their rhizosphere. In the soil they exist mainly in the form of spores, which germinate in the digestive tract of humans and animals. Germination of *Bacillus* spores in the human small intestine and transient colonization should be considered as a part of the life cycle of human-associated *Bacillus* species (Hong et al., 2009). In the GIT, spores not only germinate but also are formed again from vegetative cells during a time shorter than in the laboratory (Tam et al., 2006; Ghelardi et al., 2015).

Thus, it can be concluded that GIT microbiota including *Bacillus* and *Lactobacillus* species undergoes constant dynamic change. In our opinion, distinguishing between the resident and transient intestinal inhabitants is less relevant issue compared to the study of molecular pool released by them. Most of bacteria absorbed in the body can supplement the gastrointestinal microbiome (Derrien and van Hylckama Vlieg, 2015). The challenge of identifying the “spheres of influence” of the transient microbiota in the human body has not been solved, and has not even been formulated, with the exception of some aspects of the pathogen entry into the body. Its bottleneck is the lack of data on the complexes secreted by this microbiota, their components, functions and the interaction between components, namely, the composition and biological role of the secretome.

We consciously narrowed the spectrum of secretome producers observed here to two representatives of the *Bacilli* class, genera *Lactobacillus* and *Bacillus*, due to their wide distribution and high probability of entering the human body. The ingestion of microorganisms occurs with food, water and bacterial probiotics. Facultative aerobic bacilli represent a smaller proportion of the intestinal microbiota than anaerobic bacteria (Rajilić-Stojanović and de Vos, 2014), but they actively influence the microbial community of GIT and also the whole organism thanks to the great diversity of secreted compounds. Our studies of biopsies taken during surgical intervention in patients with diagnosed colorectal cancer revealed the presence of *Bacilli* closely associated with intestinal epithelium, traditionally considered as transient ones (Siraj et al., 2015). Since secretory components can be studied only in culturable microorganisms, the insufficiency of genomic analysis of intestinal microbiota and the transition to functional analysis became evident (Derrien and van Hylckama Vlieg, 2015; Lagier et al., 2015). In this regard, the identification, characterization, and elucidation of the functional role of the components secreted by the minority of the intestinal microbial community, namely representatives of the *Bacilli* class in GIT, is an actual task. The modern concept of a gut–brain axis (McKay et al., 2017) must be detailed and refined taking into account the spectra of compounds secreted by *Bacilli*, namely low- and high-molecular components of the secretome and EVs, which affect the whole body and shape human health.

## Beneficial Effects of *Lactobacillus* and *Bacillus*

Historically, species of *Lactobacillus* and *Bacillus* are found in the traditional fermented food products possessing beneficial properties for the intestinal function (Nithya et al., 2012; Satish Kumar et al., 2013; Lee et al., 2016; Sornplang and Piyadeatsoontorn, 2016; Marco et al., 2017) and are widely used as components of commercially available probiotics: DE111 (Deerland Enzymes, United States), Enterogermina (Sanofi Winthrop, Italy), Biosubtyl (Biophar, Vietnam), Biosporin (Biopharma, Russia), BioSpora (Klaire Labs, United States), Blicheni and Zhengchangsheng (Northeast Pharmaceutical Group, China), GanedenBC 30 (Ganeden, United States), Lactobacterin (Microgen, Russia), HOWARU or DR20 (Danisco, United States), Yakult (Yakult, Japan), PCC (Probiomics, Australia). In food industry, lactobacilli are applied as starter cultures in the production of fermented milk products, cheese, sausages, bread, kimchi, pickles, and yogurts, the latter accounting for the largest share of sales (Giraffa et al., 2010; Tamang et al., 2016). The administration of probiotics has been shown to favorably alter the intestinal microbiota balance, enhance intestinal integrity and motility, inhibit the growth of harmful bacteria and increase resistance to infections (Tamang et al., 2016).

As a part of GIT microbiota *Bacilli* participate in metabolism of dietary components, xenobiotics and drugs helping to maintain intestinal homeostasis and host health (Jandhyala et al., 2015; Rowland et al., 2017). The beneficial effect of probiotics on GIT is mediated by influence on composition, diversity and function of the intestinal microbiota as well as whole human organism. Probiotics suppress pathogenic bacteria and favor beneficial ones via competition for nutrients, especially for shared limited resource like iron, competitive attachment to the epithelium, formation of substrates for growth, production of waste products and antimicrobial compounds, strengthening of the barrier function of the epithelium, and modulation of innate immunity (Thomas and Versalovic, 2010; Bermudez-Brito et al., 2012; Stubbendieck and Straight, 2016). For example, consumption of *B. coagulans* was shown to increase beneficial groups of bacteria in the gut of 65–80 years old humans and production of anti-inflammatory cytokines (Nyangale et al., 2015).

The efficacy of *Lactobacillus* and *Bacillus* in the prevention and/or treatment of intestinal diseases such as diarrhea, colitis, irritable bowel syndrome, irritable bowel disease, and colorectal cancer was demonstrated (Camilleri, 2006; Sazawal et al., 2006; Rafter et al., 2007; Pillai and Nelson, 2008; Ghouri et al., 2014; Urgesi et al., 2014; Choi et al., 2015; Matsuoka and Kanai, 2015; Majeed et al., 2016; Zhang et al., 2016). In particular, treatment of colorectal colitis in mice with probiotic *B. subtilis* restored balance in gut microflora: beneficial species of *Bifidobacterium*, *Lactobacillus*, and *Butyricicoccus* spp. were increased, while gut damage-promoting species of *Acinetobacter* sp., *Ruminococcus* sp., *Clostridium* spp., and *Veillonella* sp. were decreased (Zhang et al., 2016). *B. subtilis* also retained gut barrier integrity, decreased the endotoxin concentration and reduced gut inflammation (Zhang et al., 2016; Bene et al., 2017). Sporulation of *B. subtilis* plays a major role in the development of GALT – gut lymphoid tissue associated with the gastrointestinal mucosa - and in the diversity of the primary antibody population (“preimmune” repertoire) in rabbits (Rhee et al., 2004). *Bacillus* spp. like other strains isolated from human stool were able to bind the human norovirus strains, the cause of acute viral gastroenteritis and foodborne diseases, around the outer cell surfaces and pili structures (Almand et al., 2017). The interaction between virus and bacteria is hypothesized to help the host immune system to better recognize infectious particles.

The ratio between the two major phyla inhabiting the human GIT, *Firmicutes* and *Bacteroidetes*, reflects the GIT status during the life and diseases. It is significantly decreased in infants and elderly individuals as compared to adults (0.4, 0.6, and 10.9, respectively) (Mariat et al., 2009) and lowers upon antibiotic-associated diarrhea, coeliac disease, Crohn’s disease, and ulcerative colitis (Ott et al., 2004; Panda et al., 2014; Carding et al., 2015; Quagliariello et al., 2016). A decrease in populations of *Ruminococcus* and *Lactobacillus* was observed in a rat model of colorectal cancer (Zhu et al., 2014). Microbial content of the patients with diagnosed colorectal cancer and healthy individuals differed significantly. *Firmicutes* and *Fusobacteria* were over-represented whereas *Proteobacteria* were under-represented in patients. In addition, *Lactococcus* and *Fusobacterium* exhibited a relatively higher abundance while *Pseudomonas* and *Escherichia–Shigella* were reduced in cancerous tissues compared to adjacent non-cancerous ones (Gao et al., 2015). *Bacilli* were shown to decrease quantitatively upon type 2 diabetes (Sankar et al., 2015). The possibility of using probiotics in the therapy of diseases, namely allergy, asthma, diabetes, cardiovascular diseases is discussed (Ebel et al., 2014).

Viability is by definition a prerequisite for probiotic effectiveness as it is essential for colonization of intestinal mucosa, displacement of pathogens and immunomodulation. Viable bacteria demonstrate adhesive and antagonistic properties and produce a large number of extracellular enzymes and biologically active compounds (Shobharani and Halami, 2014). Nevertheless, there is increasing evidence that isolated bacteria-derived molecules and surface components (e.g., cell wall components, cell wall associated proteins, S-layer proteins) potentiate probiotic benefits attributed earlier to viable probiotic bacteria (Lahtinen, 2012; Ruiz et al., 2014).

Despite more than a century of active use of probiotics, initiated by I. Mechnikov in 1907, the majority of modern reviews assessing the effectiveness of these drugs confirm the need for further studies to determine the exact mechanisms of positive effects of probiotics on the human body. Both live probiotic bacterial cells and their metabolites can be useful in treatment of intestinal diseases (Okamoto et al., 2012). The molecular basis for the effectiveness of probiotics remains unexplored or only partially studied. The future study aimed at deciphering the mechanisms that determine the probiotic properties of bacteria will certainly allow expanding the areas of scientifically proven probiotic use in medicine.

## Microbiota–Human Metabolic Interaction

Between the gut microbiota and host organism there is an extremely complex relationship that affects the human metabolism, immunity and health (Marchesi et al., 2016). This crosstalk is mediated by nutrients, metabolites, antimicrobial compounds. It was demonstrated that the psychological and physical stress of a host affects its gut microbiota and, in turn, gut microflora can modulate host’s mood and appetite (Sandrini et al., 2015). Gut microbiota is regulated by the host through production of non-specific antimicrobial peptides such as defensins (Nakamura et al., 2016), secreted IgA which provides the selection and the maintenance of the commensal bacteria (Fransen et al., 2015), and miRNAs specifically regulating bacterial transcripts and affecting bacterial growth (Liu and Weiner, 2016). It was proved that host genetic background affects the composition and function of the gut microbiota, altering the production of microbial metabolites and intestinal inflammation (Lamas et al., 2016). For example, the microbiota of mice deficient in caspase recruitment domain family member 9 (*CARD9*) failed to metabolize tryptophan that increased host susceptibility to colitis (Lamas et al., 2016).

Microbial species are recognized by host’s immune system. Commensal bacteria have immunomodulatory properties that allow them inducing tolerogenic immune responses against themselves and contributing to host protective immune responses against pathogens (Bene et al., 2017; Guo et al., 2017; Shi et al., 2017). It is known that probiotics affect key signaling pathways, such as NFκB and MAPK, through the pattern-recognition receptors (TLR, NOD) (Bermudez-Brito et al., 2012) enhancing the production of anti-inflammatory cytokines (Nyangale et al., 2015) and reducing the emergence of proinflammatory ones (Selvam et al., 2009). The gut microbiota is able to influence host antigen production by human monocyte-derived dendritic cell populations in a species-specific manner (Bene et al., 2017).

Gut microbiota affects host physiology by releasing bioactive metabolites including antibiotics, enzymes, vitamins and amino acids (choline, methionine, vitamin B), minerals (cobalt, iodine, selenium, and zinc) and energy metabolites (SAM, acetyl-CoA, NAD^+^, α-KG, and ATP), SCFAs (acetate, propionate, butyrate, caproate, and valerate), neurotransmitters [acetylcholine, dopamine, noradrenaline, serotonin, and γ-aminobutyric acid (GABA)], hormones, bacterial antigens, pathogen-associated molecular patterns, and toxins (Donia et al., 2014; Luber and Kostic, 2017). These molecules enter host circulation thereby mediating the link between the gut and other organs (brain, lung, liver, muscle) (Shukla et al., 2017) and modulate physiological pathways and even behavior (van de Wouw et al., 2017; Vuong et al., 2017). The influence of gut microbiota on the epigenetic regulation of host genes via DNA methylation and histone modifications has been demonstrated (Ye et al., 2017). Contact-independent metabolic exchange helps signal dispersal among neighboring cells as well as its blockage when needed.

**FIGURE 1:**
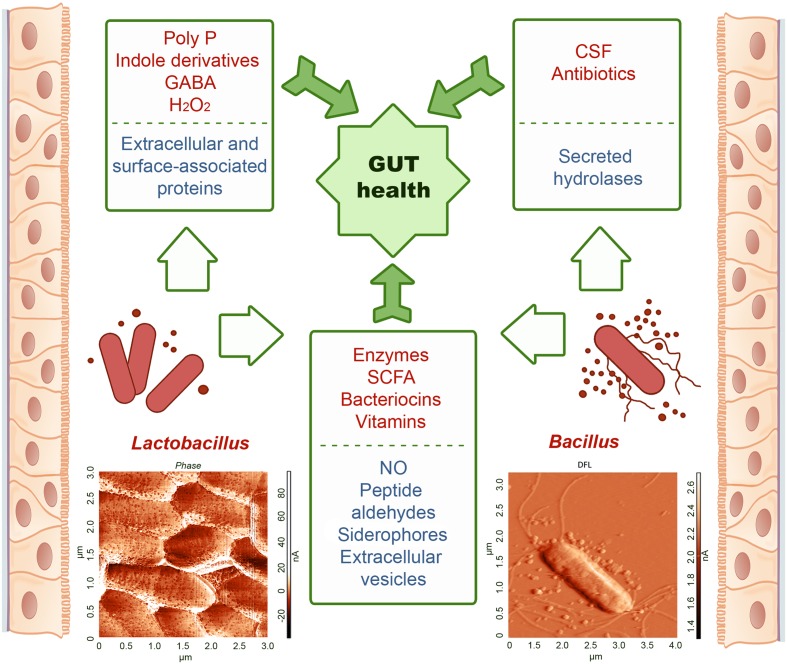
Compounds secreted by the representatives of *Lactobacillus* and *Bacillus* mediating their beneficial effects in the GIT. Top-appreciated compounds are shown in upper part of panels, under-appreciated compounds – in lower parts. Both *Lactobacillus* and *Bacillus* are minor part of GIT microbiota. Of them, *Lactobacillus* spp. dominate quantitatively secreting a few compounds, while *Bacillus* spp. are less abundant but produce a variety of secreted substances with a wide spectrum of activities. Representatives of the *Bacilli* class were isolated from colon epithelia biopsy of the patients with diagnosed colorectal cancer. Atomic force microscopy images of *L. plantarum* (photo provided by Dr. *Dina Yarullina*) and *B. pumilus* (photo kindly provided by Dr. *Galina Yakovleva*) were obtained in air (contact mode) of stationary phase cells that were deposited on glass and dried prior analysis. Bacteria were identified using MALDI-TOF technique and 16S RNA sequencing. Representative AFM images show nanoscale structures on the cell surface and around the cells attributed to EVs.

Bacteria produce a lot of chemically diverse metabolites with poorly understood function. *Bacillus* species are among the most frequent producers of bioactive secondary metabolites (800 compounds), while lactobacilli produce 100s of compounds (Bérdy, 2005). Known to date, the results of the study of representatives of the *Bacilli* class colonizing the human GIT mostly refer to ascertaining their positive, less often negative, impact on the body. We tried to systematize the available knowledge about compounds and complexes produced by these bacteria which serve as effectors triggering certain processes in the body (**Figure 1**), and identify the stage responsible for actually registered “influence.” *Bacilli* introduced into the GIT through the consumption of fermented food do integrate the resident microbiome (Derrien and van Hylckama Vlieg, 2015) and contribute to its regulatory and health promoting action producing a variety of substances ranging from low molecular weight regulatory agents to proteins and peptides with antimicrobial and antitumor effects. Metabolites secreted by bacteria form a coat around the cells which contributes to nutrient supply, communication, and protection from damage caused by direct interaction with other species or their metabolites. Due to diffusion, the concentration of extracellular metabolites decreases with distance. To ensure that secreted components will reach their targets bacteria utilize membranous vesicles for their transportation.

## Top-Appreciated Components of *Bacilli* Secretome

It is well-appreciated that complex of **enzymes** (proteases, amylases, cellulases, lipases) secreted by *Bacilli* aid in digestion of food components in GIT (Khochamit et al., 2015; Keller et al., 2017). Bile salt hydrolases of lactobacilli reduce blood cholesterol and diminish the risk for cardiovascular diseases (Patel et al., 2010; Kumar et al., 2012). Recently, an antagonistic role of *L. johnsonii* La1 extracellular bile salt hydrolase against intestinal protozoan parasite *Giardia duodenalis* was revealed (Travers et al., 2016). Other enzymes like *N*-acylated homoserine lactone (AHL)-lactonase help to modulate the microbiota content by decreasing the number of quorum-sensing pathogenic bacteria in the GIT through direct disruption of their signal molecules (Vinoj et al., 2014; Zhou et al., 2016). Moreover, many enzymes are involved in the formation of metabolites which possess their own biological activities. For instance, during fermentation of milk and other proteinaceous products lactobacilli are able to release biologically active peptides with angiotensin I-converting enzyme (ACE)-inhibitory activity. Among these antihypertensive peptides β-casein-derived tripeptides (lactotripeptides) are most studied (Hayes et al., 2007; Fekete et al., 2015).

**Short-chain fatty acids** are formed upon dietary carbohydrates fermentation by both *Bacilli* genera, studied in this review. The most common SCFA is lactate followed by acetic, propionic, butyric acids and minor isobutyrate, 2-methylpropionate, valerate, isovalerate, hexanoate. SCFAs are one of the most important gut microbial products affecting a range of host processes including energy utilization, host–microbe signaling, and control of colonic pH. Decrease of a luminal pH creates an environment favoring beneficial species like *Faecalibacterium prausnitzii* and inappropriate for many others bacteria and yeasts (Nyangale et al., 2015). SCFAs positively influence the gut motility (Yang and Chiu, 2017) and intestinal secretion (Bhattarai et al., 2017), inhibit proliferation of tumor cells by apoptosis induction, stimulate production of insulin-like growth factor 1 promoting bone growth and remodeling (Yan et al., 2016), cause epigenetic modifications, regulate blood pressure and inflammation (Natarajan and Pluznick, 2014). The multifaceted roles of SCFAs nominate them for the key molecular link between diet, the microbiome and health.

Lactate produced by *Lactobacillus* provides an unfavorable environment for the growth of many pathogenic bacteria, it also acts as a permeabilizer of the Gram-negative bacterial outer membrane, thus increasing the susceptibility of pathogens to antimicrobial molecules, e.g., bacteriocins or host lysozyme (Alakomi et al., 2000). Strains of *B. licheniformis* and *B. coagulans* also ferment different sugars with formation of lactic acid (Wang et al., 2011; Nyangale et al., 2015). Butyrate is the local energy source for colonocytes (LeBlanc et al., 2017); also it plays an important role in maintenance of the gut barrier function through stimulation of tight junction integrity and mucin production (Peng et al., 2009; Jung et al., 2015). The SCFAs produced by the human gut microbiota are transported from the gut lumen with the bloodstream to a variety of different organs, where they are used in lipid and energy metabolism, particularly by the hepatocyte cells of the liver, which use propionate for gluconeogenesis, whilst acetate and butyrate are mostly involved in lipid biosynthesis (den Besten et al., 2013a). Besides, SCFAs appear to exert regulatory effects on gluconeogenesis and lipogenesis mediated by peroxisome proliferator-activated receptor gamma (PPARγ) (den Besten et al., 2015) and protein kinases, such as AMP-activated protein kinase (Peng et al., 2009; den Besten et al., 2015) or mitogen-activated protein kinases (MAPK) (Jung et al., 2015). SCFAs have been reported to represent the natural ligands for free fatty acid receptors 2 and 3 (FFAR 2/3) (former G protein-coupled receptors, GPR43 and GPR41), involved in the regulation of lipid and glucose metabolism and found on a wide range of cell types, including enteroendocrine and immune cells (den Besten et al., 2013b). Moreover, as far as these receptors are expressed on neurons of the peripheral, autonomic and somatic nervous systems, SCFAs can modulate neuronal activity and visceral reflexes (Nøhr et al., 2015). SCFAs are considered as promising for the prevention and treatment of the metabolic syndrome, certain types of cancer, bowel disorders, such as ulcerative colitis, Crohn’s disease, and antibiotic-associated diarrhea (den Besten et al., 2013b; Ríos-Covián et al., 2016).

**Hydrogen peroxide (H_2_O_2_)** production by lactobacilli has been suggested to be a non-specific antimicrobial defense mechanism. *L. jensenii*, *L. crispatus, L. gasseri*, and *L. acidophilus* are the most common H_2_O_2_-producing lactobacilli inhabiting the human intestine and are often applied as probiotic supplements in the food industry (Martín and Suárez, 2010; Hertzberger et al., 2014). In gastrointestinal environment, SCFAs and bacteriocins have been considered as key antimicrobial factors, whereas the impact of H_2_O_2_ production remains underappreciated. However, H_2_O_2_, like other reactive oxygen species, exerts strong cytotoxicity against microorganisms. Although H_2_O_2_ itself is not highly reactive, it can readily diffuse across cellular membrane and through the Fenton reaction form highly reactive hydroxyl radicals, which cause oxidative damage to major biological macromolecules, e.g., oxidation of protein thiols, peroxidation of lipids, DNA base damage, and strand breakage of nucleic acids (Imlay, 2003; Valko et al., 2005). The role for H_2_O_2_ in the anti-*Salmonella* activity of *L. johnsonii* NCC533, the human intestinal isolate and a probiotic strain, was revealed *in vitro* (Pridmore et al., 2008). *L. delbrueckii* VI1007 produces at least three growth-inhibiting factors, other than lactic acid, one of which has been identified as H_2_O_2_ (Van De Guchte et al., 2001). H_2_O_2_ may contribute to the maintenance of the normal microbiota. Especially for the vaginal microbiota, strong evidence exists that colonization with H_2_O_2_-producing lactobacilli is associated with lower rates of bacterial vaginosis and HIV acquisition (Wilks et al., 2004; Balkus et al., 2012). Moreover, H_2_O_2_ may exert immunomodulatory properties. It was showed that H_2_O_2_, produced by *L. crispatus* M247, acts as a signal transducing molecule activating peroxisome proliferator activated receptor γ (PPAR-γ), which plays a central role in regulation of intestinal inflammation and homeostasis (Voltan et al., 2008). *L. johnsonii*-derived H_2_O_2_ has been shown to affect the activity of indoleamine 2,3-dioxygenase, an important immune modulator, both *in vitro* and in the rat model of type 1 diabetes (Valladares et al., 2013).

**Poly P,** a linear polymer of over 700 phosphate residues, is synthesized by *Lactobacillus* with the help of polyphosphate kinase (Alcántara et al., 2014). It suppresses the oxidant-induced intestinal permeability inducing cytoprotective heat shock proteins in mouse small intestine through activation of integrin β1-p38 MAPK pathway (Segawa et al., 2011). Poly P was shown to improve the inflammation grade and survival rate in mice model of colitis (Segawa et al., 2011) and to inhibit viability of colon cancer cells via apoptosis through activation of the ERK pathway (Sakatani et al., 2016).

**Indole** derivatives formed from tryptophan by *Lactobacillus* cells act on the aryl hydrocarbon receptor in intestinal immune cells increasing IL-22 production which, in turns, beneficially impacts the immune system, enhances antifungal resistance and protection of mucosa from damage (Zelante et al., 2013; Lamas et al., 2016; Etienne-Mesmin et al., 2017). The main inhibitory neurotransmitter in the brain, **GABA,** is produced with the help of glutamate decarboxylase expressed by multiple strains of *Lactobacillus* (Barrett et al., 2012; Yunes et al., 2016).

*Bacilli* synthesize B-group **vitamins** including folate and biotin during the fermentation of foods in GIT and can exchange them, thereby enabling the survival of organisms that do not synthesize those (Magnúsdóttir et al., 2015).

A number of **peptide and lipopeptide** **antibiotics and bacteriocins** are produced by *Bacilli* both involving ribosomes and non-ribosomally (Zacharofa and Lovitt, 2012; Sumi et al., 2015; Zhao and Kuipers, 2016). These structurally diverse compounds suppress the growth of competing species and pathogens through different mechanisms primarily connected to membrane permeabilization (Fiedler and Heerklotz, 2015; Shobharani et al., 2015). Antimicrobial peptides of *Bacilli* were shown to be active against pathogenic bacteria such as *Staphylococcus aureus*, methicillin resistant *S. aureus*, *Clostridium perfringens*, *Klebsiella* sp., and common food spoilage bacteria such as *B. cereus*, *Escherichia coli*, *Listeria monocytogenes, Pseudomonas aeruginosa*, *Aeromonas* sp., *Serratia marcescens*, *Pasteurella haemolytica*, *Salmonella enteritidis*, and *S. gallinarum* (Ahmadova et al., 2013; Martinez et al., 2013; Berić et al., 2014; Ayed et al., 2015; Khochamit et al., 2015; Shobharani et al., 2015; Collins et al., 2016; Lim et al., 2016; Chauhan et al., 2017; Perez et al., 2017). Bacteriocins attract great interest with regard to their potential use as food preservatives (De Vuyst and Leroy, 2007; Kaškonienė et al., 2017) and are regarded as a promising alternative to prevent gastrointestinal infections (Dobson et al., 2012).

*Lactobacillus* produces a number of bacteriocins usually active against closely related Gram-positive bacteria which are likely to reside in the same ecological niche. Most *Lactobacillus* bacteriocins are small, heat-stable cationic peptides which form pores in the cytoplasmic membrane of sensitive bacteria and thus cause leaking of target cells (Oscariz and Pisabarro, 2001). Other bacteriocins interrupt production of peptidoglycan or act by interfering with essential enzyme activities in susceptible bacteria (Servin, 2004). Bacteriocins from *Lactobacillus* are generally recognized as being inactive against Gram-negative organisms. However, it has been reported that bacteriocin from *L. plantarum* TN635 is active against *Salmonella enterica* ATCC43972, *Pseudomonas aeruginosa* ATCC 49189, *Hafnia* sp. and *Serratia* sp. (Smaoui et al., 2010). Moreover, a small bacteriocin (<6.5 kDa) produced by *L. acidophilus* IBB 801 and designated as acidophilin 801, displayed bactericidal activity against *E. coli* Row and *Salmonella panama* 1467 (Zamfir et al., 1999). Bacteriocin OR-7 produced by *L. salivarius* NRRL B-30514 resulted in reduction of *Campylobacter jejuni* colonization in chicken GI tracts when was added into feed. Interestingly, OR-7 had high sequence similarity to acidocin A, which was previously identified from *L. acidophilus* and had activity only to Gram-positive bacteria (Stern et al., 2006).

*Bacillus* is considered to be the second most important bacteriocin producer following lactic acid bacteria which differs from the latter by broad antimicrobial spectrum (Abriouel et al., 2011; Ayed et al., 2015). Bacteriocins and bacteriocin-like inhibitory substances produced by *Bacillus* exhibit antibacterial activity toward Gram-positive and Gram-negative bacteria as well as fungi, however, activity against Gram-positives is comparatively higher (Hyronimus et al., 1998; Rey et al., 2004; Arias et al., 2013; Berić et al., 2014; Chopra et al., 2014; Barbosa et al., 2015; Shobharani et al., 2015; Lee et al., 2016; Lim et al., 2016; Liu et al., 2017; Perez et al., 2017). Species of *Bacillus* differ by their antimicrobial potential (Perez et al., 2017). Non-ribosomal peptide antibiotics produced by *Bacillus* (bacitracin, proticin, lichenicidin, bacillaene) are essential for the protection of these bacteria from predation and antibiotics produced by other species (Rey et al., 2004; Barger et al., 2012; Alvarez-Ordóñez et al., 2014; Müller et al., 2014). *Bacillus* spp. were shown to produce a mixture of different lipopeptides with antimicrobial activities (Huang et al., 2006). *B. subtilis* produces surfactins, fengycins and iturins in a ratio of 6:37:57 (Fiedler and Heerklotz, 2015; Perez et al., 2017). The less abundant surfactins unlike other types of *Bacillus* lipopeptides exhibit a broad range of antimicrobial activities and possess antiviral action (Huang et al., 2006). They protect bacilli against extracellular antibiotic-containing vesicles of other species (Brown et al., 2014) and inhibit phospholipase A2 resulting in subsequent downregulation of pro-inflammatory cytokines and upregulation of anti-inflammatory cytokines (Selvam et al., 2009).

Probiotic effect of *B. subtilis* was shown to be connected to **competence and sporulation factor**, a small quorum-sensing peptide involved in bacteria communication, proliferation and sporulation (Okamoto et al., 2012). CSF activates the Akt and p38 MAPK pathways and exerts its anti-inflammatory effect by downregulation of pro-inflammatory mediators (IL-4, IL-6, and CXCL-1), the upregulation of anti-inflammatory IL-10, and the induction of cytoprotective heat shock protein Hsp27 in the intestinal epithelia (Okamoto et al., 2012). The similar effects were observed for two peptides secreted by *B. megaterium* isolated from human ileal biopsies of healthy volunteers (Di Luccia et al., 2016). Effects of CSF depend on its uptake by an organic cation transporter-2 in intestine which helps the host to monitor and respond to changes in the behavior or composition of colonic microbiota (Fujiya et al., 2007).

## Under-Appreciated Components of *Bacilli* Secretome

**Extracellular and surface-associated proteins** secreted by commensal bacteria play an important role in gut colonization and persistence. Moreover, some of them can interact directly with mucosal cells, activating signaling pathways that lead to different cytokine secretion and gene expression profiles (Tsilingiri and Rescigno, 2013). For instance, two secreted proteins p75 and p40 (also known as Msp1 and Msp2) of *L. rhamnosus* GG have been demonstrated to prevent cytokine-induced cell apoptosis by activating the antiapoptotic protein kinase B and by inhibiting the pro-apoptotic MAPK (Yan and Polk, 2002; Yan et al., 2007), reduce TNF induced epithelial damage in the colon and as a result promote epithelial homeostasis (Yan et al., 2007). Homologs of genes that encode for p40 and p75 were also found in the genomes of *L. casei* and *L. rhamnosus*; the proteins from *L. casei* BL23 were demonstrated to elicit similar host responses (Bäuerl et al., 2010).

**Secreted hydrolytic enzymes** contribute to probiotic effects of *Bacilli* due to their ability to decompose food polymers releasing digestive discomfort. However, accumulating data indicate that these proteins might be involved in a complex interaction with host and its microbiota. Hydrolases demonstrate direct antimicrobial activity. Proteases, glycoside hydrolases and DNases participate in dispersal of bacterial biofilms and inhibition of biofilm formation (Chen et al., 2013; Nguyen and Burrows, 2014; Watters et al., 2016; Fleming and Rumbaugh, 2017). Extracellular nuclease, NucB, from *B. licheniformis*, was shown to digest extracellular DNA in biofilms of staphylococci and streptococci associated with chronic rhinosinusitis proving enzyme effectiveness in eradicating biofilms of multidrug-resistant bacteria (Shields et al., 2013). Secretion of low-molecular-weight guanyl-preferring ribonucleases (RNases) is a distinct feature of some *Bacillus* species (Ulyanova et al., 2016). A well-studied representative of these RNases, binase from *B. pumilus*, has manifested antitumor (Ulyanova et al., 2011; Cabrera-Fuentes et al., 2013; Mitkevich et al., 2013) and antiviral activities (Shah Mahmud and Ilinskaya, 2013; Ilinskaya and Shah Mahmud, 2014; Shah Mahmud et al., 2016, 2017; Müller et al., 2017). KRAS which has mutations in about 40% of patients with colorectal cancer (Prior et al., 2012) was shown to be a direct target for antitumor binase (Ilinskaya et al., 2016). 2′,3′-cGMP generated by binase upon RNA cleavage (Sokurenko et al., 2015) may also contribute to modulation of host cells physiology, since it is able to duplicate its counterpart 3′,5′-cGMP (Boadu et al., 2001) and can exhibit its own regulatory functions. Extracellular cGMP enhances extracellular adenosine and reduces uric acid levels which may render tissue protective effect upon injury (Jackson et al., 2013). Guanylate cyclase which catalyzes the synthesis of cGMP from GTP is represented in the cell membranes along the intestine. The cGMP signaling regulates intestinal fluid and electrolyte balance, epithelial homeostasis, mucosal barrier integrity, visceral sensation through ERK and AKT pathways (Han et al., 2011; Lin et al., 2012; Hannig et al., 2014; Lan et al., 2016). Increased cGMP levels in the colon epithelium activate antioxidant gene expression (Wang et al., 2017). cGMP expression is significantly decreased upon ulcerative colitis and colon cancer (Lin et al., 2012; Lan et al., 2016; Pattison et al., 2016). cGMP and ways for enhancement of its production are considered for treatment of irritable bowel syndrome by decreasing of gastrointestinal pain and abdominal sensory symptoms (Lan et al., 2016) and as a tool for tumor suppression (Pattison et al., 2016).

Recently, gut *Firmicutes* were shown to produce **peptide aldehydes**, cell-permeable protease inhibitors with a half-life of hours, which target cathepsins in the host lysosome blocking immune recognition of these mutualistic species and enabling them to reside in gut epithelial (Guo et al., 2017).

**Nitric oxide** is a well-known ubiquitous molecular mediator produced in mammals by the NOS isoforms at a catalytic site comprising a heme associated with a biopterin cofactor. Genome sequencing has shown the presence of genes encoding for proteins that are highly homologous to the oxygenase domain of mammalian NOS in bacteria, including those of the class *Bacilli*: *S. aureus* (Bird et al., 2002; Salard et al., 2006), *B. subtilis* (Adak et al., 2002), *B. anthracis* (Midha et al., 2005; Salard et al., 2006), *Geobacillus stearothermophilus* (Sudhamsu and Crane, 2006), *L. fermentum* (Morita et al., 1997), and *L. plantarum* (Adawi et al., 1997; Iarullina et al., 2006; Iarullina and Ilinskaia, 2007; Yarullina et al., 2015). So, intestinal *Bacilli* have NOS that is evolutionary related to the mammalian enzymes. Moreover, as bacteria have the most ancient version of NOS, it was hypothesized that Eukaryotes acquired NOS from bacteria by horizontal gene transfer (Gusarov et al., 2013). Recently, the conservation of NOS-derived NO-heme receptor signaling between bacteria and mammals was proved (Kinkel et al., 2016). NO as reactive oxygen molecule is widely considered as important participant in the immune system of different organisms to confront microbial infections. Thus, inhibition of bacterial NOS has the potential to improve the efficacy of antimicrobials used to treat infections by Gram-positive pathogens *S. aureus* and *B. anthracis* possessing this enzyme (Holden et al., 2015). Commensal microbiota-derived NO has been shown to influence host physiology. NO synthesized by *L. plantarum* takes part in the regulation of intestinal motility in rat (Yarullina et al., 2016). Being a signaling molecule, NO released by *B. subtilis* in the intestine of *Caenorhabditis elegans* initiates a signaling cascade that results in the induction of 65 genes, including *hsp*s and several other genes that have been implicated in longevity and stress resistance (Gusarov et al., 2013). Involvement of bacterial NO in human cardiovascular system is under investigation (Cabrera-Fuentes et al., 2016).

**Ferrichrome** of *L. casei* was identified as a tumor-suppressive molecule on colon cancer cells which induces apoptosis via activation of c-jun N-terminal kinase (JNK) (Konishi et al., 2016). Many siderophore-binding proteins were found in EVs of *B. subtilis* (Dubois et al., 2009; Brown et al., 2014). Siderophores can endow bacilli advantage in competition for low-available iron with pathogenic bacteria. Iron cations are potent crosslinkers of the biofilm matrix (Chen and Stewart, 2002) and their chelation causes dispersal of biofilms (Sobke et al., 2012).

Over the last decade, **extracellular vesicles** have emerged as prominent vehicles of biological signals. Intense research on that topic revealed that EVs play important roles in bacterial physiology and pathogenesis, ranging from secretion and delivery of biomolecules (for example, toxins, DNA, or quorum sensing molecules) over stress response and biofilm formation to immunomodulation and adherence to host cells (Roier et al., 2016). Both *Bacillus* and *Lactobacillus* species were reported to produce EVs, spherical membranous structures of 20–150 nm in diameter (Brown et al., 2014, 2015; Avila-Calderón et al., 2015; Behzadi et al., 2017; Li et al., 2017). EVs are formed both in planktonic cultures and bacterial biofilms where they help to maintain biofilm cohesion. The quantity of EVs varies with the strain (Brown et al., 2014), conditions and stage of growth (Kim et al., 2016). Thus, EVs can be produced by Bacilli in GIT.

The EVs are enriched with proteins, lipids, nucleic acids, and metabolites which exhibit biological activities. Release of vesicular cargo is achieved by direct intercellular transfer mediated by the membranes fusion (Kim et al., 2016; Stubbendieck and Straight, 2016) or by production of special molecules like lipopeptide surfactin which disrupts EVs unspecifically (Brown et al., 2014). Therefore, proteins with specific biological activities can be directly delivered inside EVs into other cells ensuring their penetration. In Gram-positive bacteria, proteins secreted via specific pathways are believed to be important for nutrient acquisition, detoxification, competitive survival, and communication (Brown et al., 2015). Recent findings support the importance of EVs for interaction of bacteria with each other and the host cells (Kim et al., 2016).

Extracellular vesicles carry hydrolytic enzymes for nutrient acquisition from extracellular complex substrates or key nutrients to feed sibling cells and contain specific agents for antagonizing competing species. In EV important for survival compounds are protected from damage retaining activities much longer and can be transported in concentrated amounts for long distances from producing cells. Among these compounds are antibiotics and hydrolytic enzymes including peptidoglycan-degrading hydrolases (Mashburn and Whiteley, 2005; Alves et al., 2016; Stubbendieck and Straight, 2016). EVs isolated from *B. subtilis* contain proteins which are mostly associated with metabolic pathways including biosynthesis of secondary metabolites (Brown et al., 2014; Kim et al., 2016). Proteins with oxidoreductase and nucleotide binding activities are abundant in vegetative EVs, while proteins with hydrolytic, nucleic acid binding, and structural activity are predominant in sporulating EV (Kim et al., 2016). In EVs of sporulating *B. subtilis* superoxide dismutase, alkaline phosphatase III, polyketide synthase PKsM (associated with antibiotic activity) were identified (Kim et al., 2016). Sunl protein which confers self-immunity to antibiotic sublancin and many siderophore-binding proteins were found in EVs of *B. subtilis* (Dubois et al., 2009; Brown et al., 2014). The targeted lysis of EVs by surfactin of *B. subtilis* is hypothesized to guard bacilli from alien EVs and disrupt cell signaling by means of EVs in competing populations (Stubbendieck and Straight, 2016). EVs were also shown to adsorb phages (Biller et al., 2014).

Interaction of EVs with the host is specific to the microorganism from which the EVs were produced and is based on the lipid content and cargo of the EVs (Brown et al., 2015). Gram-positive bacterial EVs are composed of various fatty acids which might have a positive effect on host organism (Rivera et al., 2010). EVs were shown to elicit protective immune response in host (Vargas et al., 2015). For example, treatment of *C. elegans* with EVs originated from *L. plantarum* WCFS1 led to increased transcription of host defense genes, *cpr-1* and *clec-60*, and thus provided protection against vancomycin-resistant *Enterococcus faecium*. Moreover, in human Caco-2 cells these EVs had similar effect, leading to the upregulation of *REG3G*, which is functionally similar to *clec-60*, and *CTSB*, the human ortholog of *cpr-1* (Li et al., 2017). The EVs from *B. lentus* isolated from Korean soybean fermented food induced apoptosis of human colon carcinoma cells HCT116 (Yang et al., 2016). EVs derived from *L. rhamnosus* GG are likely to be implicated in the anti-cancer activity as they induce apoptosis in the hepatic adenocarcinoma cell line HepG2 via augmentation of the expression ratio between pro- and anti-apoptotic genes *bax/bcl-2* (Behzadi et al., 2017).

## Conclusion and Further Perspectives

Now, it has become clear that studies on phylotype profiling are limited to the identification of microbial constituents, where information is lacking about the molecular interaction of bacterial communities with the host. Lactobacilli are well-represented in the human GIT and secrete a number of compounds which have direct and indirect effects on the health of GIT and organism as a whole. Species of *Bacillus* genus are much less abundant but are capable of producing several times more extracellular molecules than lactobacilli. Many of them still require exploration. Further deep studies are needed for better understanding of the complex interactions between human organism and its microbiota, clarification of the particular mechanisms underlying remarkable beneficial properties of probiotic *Bacilli*, and the specific action of innumerous secreted low- and high-molecular weight compounds and their vesicular transportation.

## Author Contributions

The basis of this review was the experimental work of the group led by ONI, in which a variety of biological activities of bacilli and lactobacilli secretome was established. ONI developed the main idea of this review, collected literature data, verified the text and coordinated it with co-authors carrying out the general guidance of the review. The part of the review devoted to the properties of representatives of the genus *Bacillus* and the components of their secretome belongs to VVU. She structured the review and created the scheme summarizing the main idea of the review on the diversity of components secreted by the representatives of Bacilli class. DRY, being a specialist in the field of probiotic activity of lactobacilli, analyzed data on the influence of *Lactobacillus* and their metabolites on intestinal functions and microbiota, and host organism. IGG collected literature data on the microflora of the human intestine. On the basis of his analysis of epithelial biopsy samples obtained during the operations of patients with diagnosed colorectal cancer, a conclusion about the contribution of Bacilli class to the microbiota closely associated with the epithelium was made.

## Conflict of Interest Statement

The authors declare that the research was conducted in the absence of any commercial or financial relationships that could be construed as a potential conflict of interest.

## Abbreviations

CSF: competence and sporulation factor
EVs: extracellular vesicles
GIT: gastrointestinal tract
NO: nitric oxide
NOS: nitric oxide synthase
RNases: ribonucleases
SCFAs: short-chain fatty acids
